# Integrative Analyses of Metabolome and Transcriptome Reveal Scion–Stock Asymmetry Reduction and Shift of Sugar Metabolism During Graft Junction Formation in Malus Domestica (‘Hanfu’) Homograft

**DOI:** 10.3390/ijms26115290

**Published:** 2025-05-30

**Authors:** Wenting Huang, Shengyuan Wang, Chong Mao, Ling Xiang, Xiao Zhang, Feng Jiang, Yuqin Cheng, Tianzhong Li

**Affiliations:** College of Horticulture, China Agricultural University, No. 2 Yuanmingyuan West Road, Haidian District, Beijing 100193, China; cauwthuang@foxmail.com (W.H.); wsy0515@yeah.net (S.W.); mcsixa@163.com (C.M.); xiangling127@163.com (L.X.); zhangxiao@cau.edu.cn (X.Z.); jiangfeng@cau.edu.cn (F.J.); chengyuqin@cau.edu.cn (Y.C.)

**Keywords:** apple grafting, vascular tissue regeneration, metabolome, transcriptome, soluble sugar

## Abstract

Grafting is widely used as a breeding method to enhance productivity and resilience. However, the mechanisms of graft healing remain poorly understood. In this study, we performed Malus domestica (‘Hanfu’) homograft and observed morphological and anatomical changes during the healing process in the graft junction within 40 days after grafting (DAG). The results showed that the healing process was divided into two phases: 0–20 days (callus proliferation phase) and 20–40 days (vascular bundle reconnection phase). During the early stage (20 DAG), gene expression exhibited asymmetry between the scion and rootstock, whereas synchronization occurred in the late stage (40 DAG). Transcriptomic and metabolomic analyses of the scion and rootstock during these two critical phases identified that differentially expressed genes (DEGs) were enriched in “Carbon fixation by Calvin cycle” and “photosynthesis-related pathways”, while differentially expressed metabolites (DEMs) were clustered in “Galactose metabolism”, implying a critical role of carbohydrates in grafting. Genes encoding enzymes involved in sugar biosynthesis, such as amylase (MdAMY), invertase (MdINV), galactinol synthase (MdGS), raffinose synthase (MdRS), and stachyose synthase (MdSS), were generally more highly expressed during Phase I than Phase II. In contrast, genes encoding enzymes related to sugar consumption, such as fructose kinases (MdSUS), cellulose synthases (MdCESA), and galacturonosyltransferase (MdGAUT), showed weak expression in Phase I but were strongly activated in Phase II. Glucose, sucrose, galactose, and melibiose levels increased significantly at 20 DAG compared with 0 DAG and subsequently decreased by 40 DAG. Exogenous application of 0.5% sucrose, raffinose, or melibiose significantly enhanced vascular bundle reconnection rates at 7 DAG compared with the control group (*p* < 0.01), confirming the pivotal role of sugar metabolism in graft healing.

## 1. Introduction

Grafting is one of the most commonly used asexual reproduction methods in plants, involving the attachment of organs or tissues from one plant to another plant at an appropriate site, allowing the two to heal into a new plant. Grafting technology is widely used in the cultivation of vegetables, fruit trees, flowers, and forestry, as well as in research areas such as plant physiology, pathology, virology, and organogenesis [1]. Additionally, it plays important roles in preserving germplasm resources, improving production efficiency, enhancing crop quality, controlling growth and development, and increasing stress resistance [2]. Apple asexual propagation is almost exclusively carried out through grafting to enhance resistance to biotic and abiotic stresses. However, there are often instances of plant death, wind breakage, and slow growth after grafting, which are mainly caused by differences in genetic background, grafting environment, and grafting techniques [3], with genetic background differences being the most significant. It is generally believed that the closer the kinship between scion and rootstock, the stronger the grafting compatibility [4].

The process of graft junction formation is usually divided into three stages [5]. (1) After mechanical damage, the broken cell tissues at the graft interface of the rootstock and scion form an isolation layer, which is considered a universal response of plants to mechanical damage and is unrelated to graft compatibility [6]. (2) The isolation layer gradually disappears as callus tissue forms and proliferates. Once the callus tissues from the scion and rootstock come into contact and merge, they form a callus tissue bridge that facilitates material exchange and cell-to-cell communication [7]. Plasmodesmata-associated genes were observed to be upregulated immediately after callus adhesion, which may provide a link to symplasm establishment [8]. Plasmodesmata is the basis for the further differentiation of the cambium into xylem and phloem and the reconnection of the vascular bundles [9]. (3) The callus tissue re-differentiates into xylem and phloem and other conductive tissues, establishing a vascular bundle bridge between the scion and rootstock, enabling the exchange of water, inorganic salts, and macromolecules [9].

Research on grafting healing mechanisms and compatibility has identified key molecular components participating in the transition from wound signaling to vascular reconnection between rootstock and scion in Arabidopsis [10]. Transcriptomic analyses across diverse plant species, including grapevines [11], lychee trees [12], and hickory trees [13], reveal extensive transcriptional reprogramming during grafting, inducing thousands of differentially expressed genes. Most graft-induced genes are involved in wound response, hormone response, signal transduction, and cell wall synthesis [14,15]. Notably, asymmetric expression patterns between the rootstock and scion are frequently observed, particularly in carbohydrate-responsive genetic networks, which may be related to the accumulation of starch above the graft union in the phloem tissue [16]. The accumulation of sugars in the scion and the depletion of sugars in the rootstock can lead to asymmetric expression levels of sugar-induced genes such as ADP-glucose pyrophosphorylase (ApL3), dark inducible 6 (DIN6), glutamate dehydrogenase 1 (GDH1), and sugar transporter protein 1 (STP1). This asymmetric gene expression is a critical feature of grafting and tissue healing and may play an important role in vascular connections at the graft union [16]. β-1,4-glucanase, encoded by the glycosyl hydrolase gene GH9B3, has been shown to be secreted into the extracellular region during the grafting process in tobacco, altering the cell wall structure to facilitate contact and signal exchange between rootstock and scion cells, enabling successful grafting between Nicotiana species and a diverse range of angiosperms [17]. Sugar transporters SWEET15 and SWEET19 have been shown to activate cell-to-cell communication between scion and rootstock cells [16]. Although the role of sugars in vascular formation is not fully established, sugars are known to promote cell division and expansion [18], such as sucrose affecting the quantity of callus tissue on sieve plates and further promoting the formation of vascular bundles, indicating that the formation of phloem and xylem requires the presence of sugars [19]. Adding 0.5% sucrose to the grafting medium can accelerate recovery after grafting and increase graft success rates [20].

In this study, Malus domestica (‘Hanfu’) homograft was performed to examine changes in gene expression and metabolite profiles during the whole process of graft junction formation in order to identify genes and metabolism involved in this process, highlighting genes and metabolites in different stages in order to construct transcriptional and metabolic regulation networks in graft junction formation.

## 2. Results

### 2.1. The Process of the Graft Junction Formation

**Figure 1:**
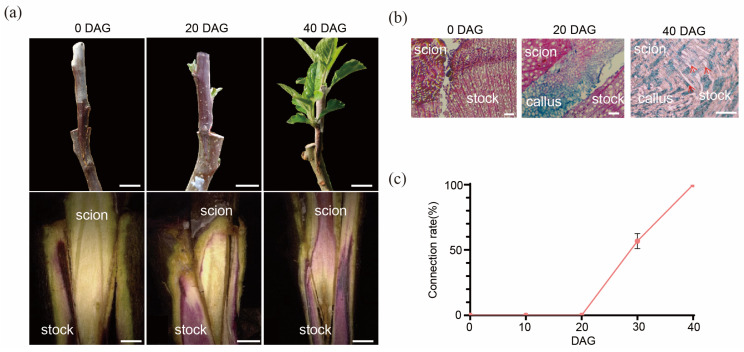
The phenotype of ‘Hanfu’ homograft. (**a**) The upper panel shows the growth condition of the grafted plants at 0, 20, and 40 DAG (the scale bar in the image represents 1 cm in length). The lower panel shows the results of xylem connectivity assessed by acid fuchsin staining (the scale bar in the image represents 2 mm in length). (**b**) Anatomic changes at the graft junction site. Arrows indicate vascular bundles connecting the scion and the stock (the scale bar in the image represents 200 μm in length). (**c**) Connection rate of the grafted plants at different stages.

The survival rate of the ‘Hanfu’/‘Hanfu’ homograft was 100% at 40 DAG. After grafting, it can be observed that callus tissue begins to form and gradually increases to fill the gap between the rootstock and scion within 20 days. The dormant buds of the scion began to germinate at 20 DAG. Acid fuchsin, fed from the stock end to monitor the reconnection of the xylem, was not observed in the scion end at 20 DAG, while all the scions were stained with red dye at 40 DAG, indicating that even though the scion has started to sprout, the vascular was not connected (Figure 1a). The anatomical results indicate the differentiation of vascular tissue from the callus and the first vascular connection between the stock and the scion at 20 DAG (Figure 1b), after which the vascular connection rate increased significantly. Callus formation from the scion could be observed at the grafting junction, and the callus filled up the graft junction, bridging the scion and the stock by 20 DAG. Vascular reconnection started after 20 DAG and was fully connected at 40 DAG (Figure 1c) when numerous vascular bundles bridging the stock and the scion could be observed.

### 2.2. RNA Sequencing and Functional Annotation of DEGs

Samples were collected as described (Figure S1). A total of 18 samples were tested in this analysis, 90.16 Gb of data was obtained, and the percentage of Q30 bases was 95.25% or above. Raw data obtained by sequencing were filtered, and clean reads obtained were compared to the reference sequence. Sequence alignment between CleanReads of each sample and the HFTH1 reference genome [21] was performed using Histaq2. The matching rate of alignment ranged from 93.88% to 96.66%. After matching the sequenced reads to the reference genome and reconstructing transcripts, a total of 2701 new genes were detected, of which 2543 were functionally annotated.

**Figure 2:**
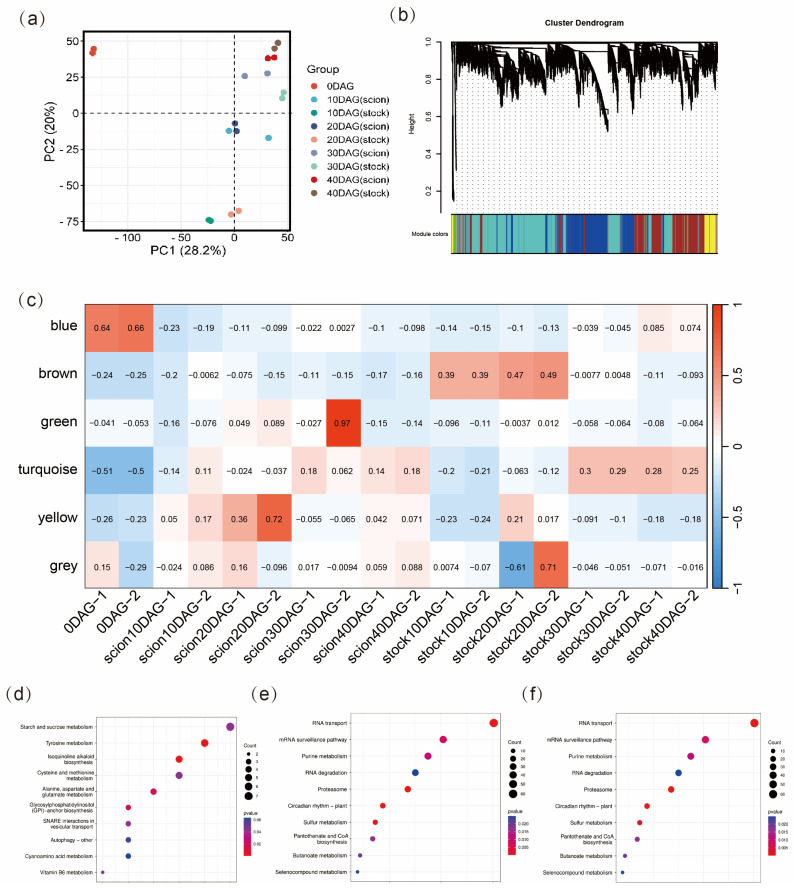
Principal component analysis (PCA) and weighted correlation network analysis (WGCNA) based on the transcriptomic data from 18 samples. (**a**) Principal component analysis (PCA) of the samples. (**b**) Cluster dendrogram showing co-expression modules (clusters) identified by WGCNA. (**c**) Heat map showing module-stage correlations for both the stock and the scions. (**d**–**f**) KEGG enrichment for genes in (**d**) yellow, (**e**) brown, and (**f**) turquoise modules.

A principal component analysis (PCA) was performed to visualize the transcriptome trend of the 18 samples analyzed. The first two principal components defined 28.2% and 20% of the variance among samples, respectively (Figure 2a). PCA results showed great transcriptomic changes after grafting. In the PCA score plot, samples at 0 DAG with the lowest PC1 scores are distant from the other samples. The stock and the scion samples at 10 DAG and 20 DAG are distant, while they are close at 30 DAG and 40 DAG. These results indicate that there is a clear asymmetry in gene expression patterns between the rootstock and scion during the early stages of the grafting process, which involves callus tissue differentiation and proliferation, while gene expression patterns became similar in the later stages, which involves vascular reconnection.

### 2.3. Weighted Gene Co-Expression Network Analysis (WGCNA)

To gain further insight into the transcriptomic changes throughout grafting, WGCNA was conducted to gain co-expression of genes. Six modules of genes were identified (Figure 2b,c, Table S1). The yellow module contained 476 genes, which were highly expressed in the early stages (0–20 DAG) in the scion but relatively weakly expressed in the stock. The brown module had 2376 genes, which were more active in the stock in the early stage (0–20 DAG) of graft junction formation. The 4272 genes in the turquoise module were highly expressed in the late stage (20–40 DAG) of graft junction formation both in the scion and the stock. To further understand the roles of these genes, KEGG enrichment analyses were applied to the three modules.

The most enriched pathways in the yellow module included ‘starch and sucrose metabolism’, ‘tyrosine metabolism’, ‘isoquinoline alkaloid biosynthesis’, and ‘amino acid metabolism’, while the main enriched pathways in the brown module included ‘spliceosome’, ‘RNA degradation’, ‘oxidative phosphorylation’, and ‘peroxisome’. The result again suggested that the scion and the stock cells around the graft junction differed greatly in the transcriptome. The main enriched pathways in the turquoise module, with genes displaying an increasing expression pattern in both the stock and the scion, included ‘RNA transport’, ‘mRNA surveillance pathway’, ‘purine metabolism’, ‘RNA degradation’, and ‘proteasome’, suggesting activation of gene translation and error-correcting functions in translation during graft junction formation in both the stock and the scion (Figure 2d–f).

### 2.4. Pairwise Transcriptomic Comparison Between Stages of Graft Junction Formation

**Figure 3:**
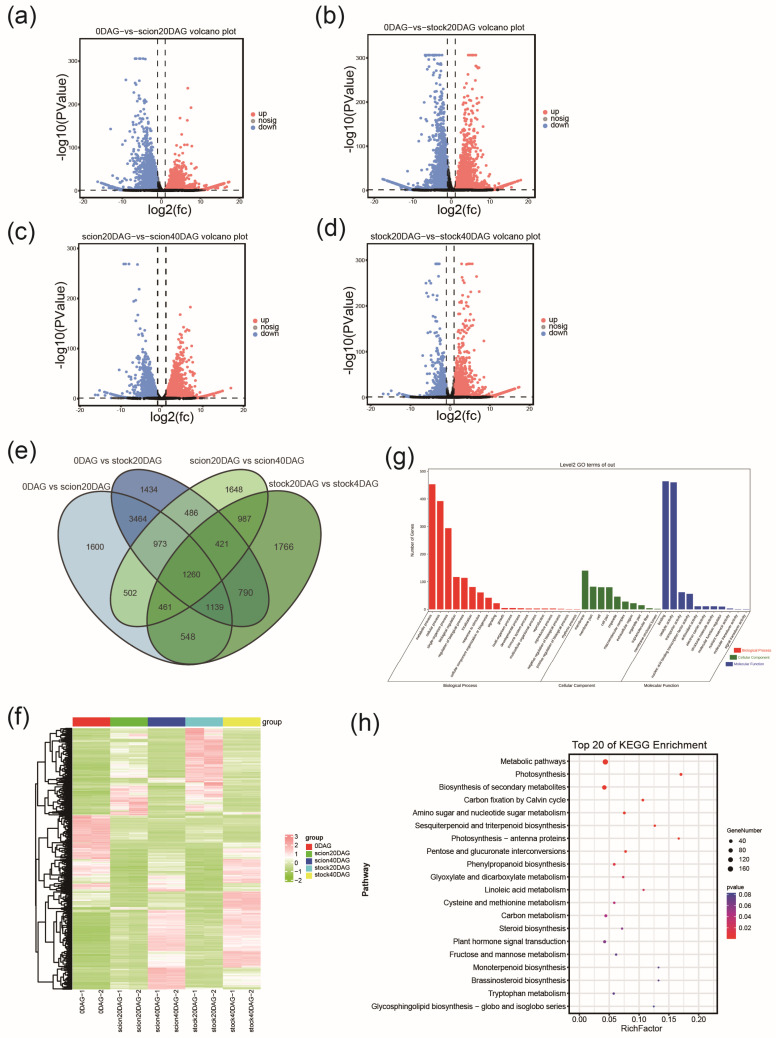
Transcriptomic comparison and KEGG enrichment of differentially expressed genes (DEGs) between stages. (**a**–**d**) Volcano plots for DEGs in the comparisons of 0 DAG vs. 20 DAG (scion), 0 DAG vs. 20 DAG (stock), 20 DAG vs. 40 DAG (scion), and 20 DAG vs. 40 DAG (stock). (**e**) Number of DEGs in pairwise comparisons. The horizontal axis shows sample names and the results of hierarchical clustering, while the vertical axis displays DEGs and their corresponding clustering results. Red indicates high expression, and green indicates low expression. (**f**) Venn diagram analysis of DEGs during grafting. (**g**) Heatmap of clustered 1260 DEGs. (**h**) KEGG pathway assignment of 1260 DEGs.

To identify the key genes involved in the stock and scion at early and late stages of graft junction formation, we selected samples at 0, 20, and 40 DAG for paired transcriptomic comparisons, including 0 DAG vs. 20 DAG (scion), 0 DAG vs. 20 DAG (stock), 20 DAG vs. 40 DAG (scion), and 20 DAG vs. 40 DAG (stock). Differentially expressed genes (DEGs) were identified according to their expression levels in different samples. DEGs were screened at a criterium of |log2FC| > 1 and *p* < 0.05 (Table S2). A total of 9947 (7406 upregulated and 2541 downregulated), 9967 (7173 upregulated and 2794 downregulated), 6738 (3825 upregulated and 2913 downregulated), and 7372 (4398 upregulated and 2974 downregulated) DEGs were screened out in 0 DAG vs. 20DAG (scion), 0 DAG vs. 20 DAG (stock), 20 DAG vs. 40 DAG (scion), and 20 DAG vs. 40 DAG (stock), respectively (Figure 3a–d). More DEGs were found in the early stages than in the late stages in both parts. Venn diagrams of differentially expressed genes showed that 1260 DEGs were identified across all comparisons. A hierarchical clustering analysis was conducted on the combined set of 1260 DEGs, and a clustering heatmap was generated for each differential group. The results revealed that samples from the same stage were clustered into one subclass (Figure 3f). To further understand the functional roles of the identified DEGs, we performed GO enrichment analysis. The GO enrichment analysis revealed that these 1260 DEGs are primarily concentrated in the biological process terms ‘metabolic process’ and ‘cellular process’, the molecular function terms ‘binding’ and ‘catalytic activity’, and the cellular component term ‘membrane’. These results suggest that the DEGs are involved in key metabolic and cellular functions within the studied system (Figure 3g). The major KEGG-enriched pathways of the 1260 DEGs included ‘metabolic pathways’, ‘carbon fixation by Calvin cycle’, ‘photosynthesis-related pathways’, and pathways of secondary products, including flavonoids, steroids, and terpenoids (Figure 3h). This implies that graft junction formation involves considerable changes in carbohydrates and that sugar metabolism may play a significant role in the process of graft junction formation.

### 2.5. Changes in Metabolite Profiling During Graft Junction Formation

**Figure 4:**
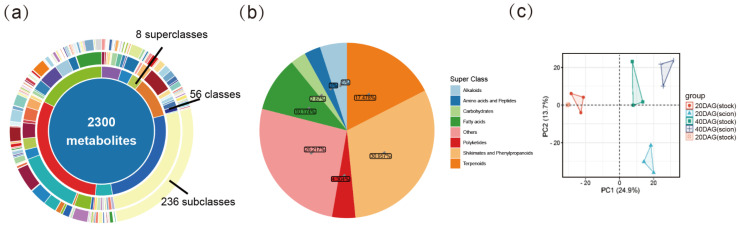
Metabolomic analysis of the scion and the stock around the graft junction at different stages. (**a**,**b**) Clustering of the metabolites identified. (**c**) PCA plot of variations.

We performed metabolomic analysis on samples of the scion and the stock at 3 crucial stages (0, 20, and 40 DAG). A total of 2300 metabolites were identified after relative standard deviation de-noising. They could be categorized into 8 superclasses, 56 classes, and 236 subclasses. Among them, 30.95% were identified as shikimates and phenylpropanoids, 17.48% were identified as terpenoids, and 10.17% were identified as fatty acids (Figure 4a,b, Table S3). PCA was carried out to visualize the distribution and the grouping of the samples (Figure 4c). Similar to the results of PCA based on transcriptomic data, the sample points of the scion at 20 DAG were distant from those of the stock at 20 DAG, the latter being very close to samples at 0 DAG. The sample points of the scion and the stock became closer at 40 DAG compared with 20 DAG, and both shifted toward higher scores on PC1 and PC2. The results indicate that there was a great difference in metabolite profiles between the stock and the scion at 20 DAG, while their metabolomic difference was reduced at 40 DAG. Again, the results suggest a shift from asymmetry to relative symmetry in metabolism between the stock and the scion during the process of graft junction formation.

**Figure 5:**
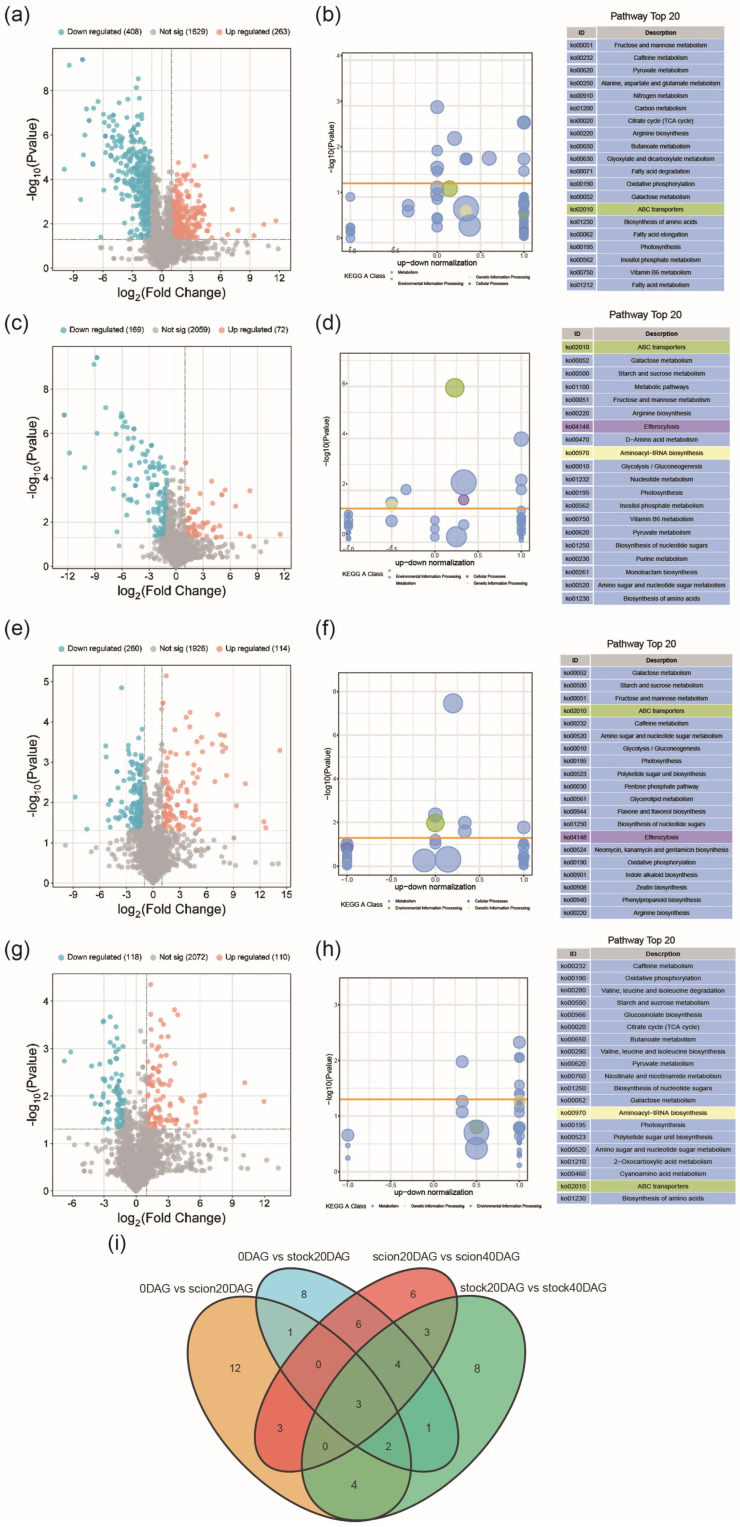
Metabolomic comparison and pathway analyses of differentially expressed metabolites (DEMs) between stages. (**a**,**b**) DEMs and pathway analysis for 0 DAG vs. 20 DAG (scion). (**c**,**d**) DEMs and pathway analysis for 0 DAG vs. 20 DAG (stock). (**e**,**f**) DEMs and pathway analysis for 20 DAG vs. 40 DAG (scion). (**g**,**h**) DEMs and pathway analysis for 20 DAG vs. 40 DAG (stock). (**i**) Venn diagram of the top 25 KEGG-enriched pathways for all comparison groups.

To explore changes in metabolomic profiles during graft junction formation, we investigated the differentially expressed metabolites (DEMs) between samples at different stages. An orthogonal partial least squares discriminant analysis (OPLS-DA) demonstrated clear separation between control and treatment groups, with high R2Y and Q2 values for the model, indicating good predictive ability (Figure S2). OPLS-DA model analysis with the selection criteria set as VIP > 1 and *p*-value < 0.05. 671, 241, 374, and 228 DEMs were identified for 0 DAG vs. 20 DAG (scion), 0 DAG vs. 20 DAG (stock), 20 DAG vs. 40 DAG (scion), and 20 DAG vs. 40 DAG (stock), respectively. Among them, 263, 72, 114, and 110 were upregulated, while 408, 169, 260, and 118 were downregulated, respectively (Figure 5a,c,e,g, Table S4).

KEGG enrichment of the DEMs was carried out for the DEMs. In 0 DAG vs. 20 DAG (scion), DEMs were highly enriched in ‘galactose metabolism’, ‘Alanine aspartate and glutamate metabolism’, and ‘fructose and mannose metabolism’ (Figure 5b). In 0 DAG vs. 20 DAG (stock), DEMs were highly enriched in ‘galactose metabolism’, ‘starch and sucrose metabolism’, and ‘fructose and mannose metabolism’ (Figure 5d). In 20 DAG (scion) vs. 40 DAG (scion), DEMs were highly enriched in ‘galactose metabolism’, ‘fructose and mannose metabolism’, and ‘starch and sucrose metabolism’ (Figure 5f). In 20 DAG vs. 40 DAG (stock), DEMs were highly enriched in ‘oxidative phosphorylation’, ‘caffeine metabolism’, and ‘starch and sucrose metabolism’ (Figure 5h). The top 25 KEGG-enriched pathways for all comparison groups were intersected, and the Venn diagram revealed that three pathways were significantly enriched across all comparison groups: ‘metabolic pathways’, ‘ABC transporters’, and ‘galactose metabolism’(Figure 5i). It can be found that there were significant changes in pathways of sugar metabolism during graft junction formation in both the stock and the scion, which again indicates that sugars may have a physiological role in graft junction formation. Therefore, we examined the details of gene expression profiles of enzymes and metabolites involved in the sugar metabolism pathway.

### 2.6. Sugar Metabolism Shift During Graft Junction Formation

**Figure 6:**
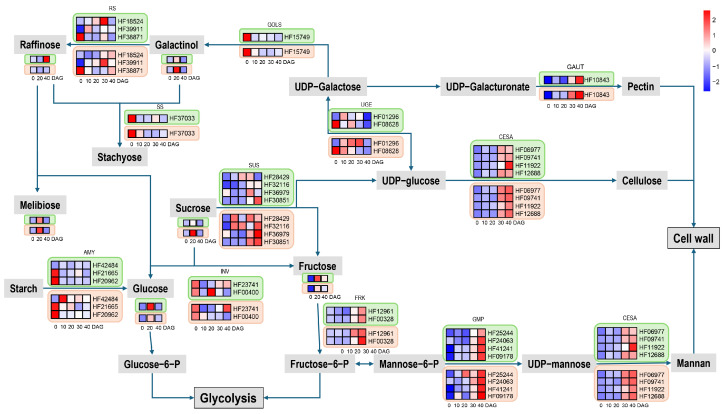
Gene expression profiles of enzymes and metabolites involved in sugar metabolism pathway. Green-framed heatmaps indicate genes or metabolites expressed in the scion, while beige-framed heatmaps indicate those expressed in the stock.

Key genes and metabolites involved in sugar metabolism were identified with differential expressions. Genes of enzymes catalyzing sugar generation, such as amylase (MdAMY), invertase (MdINV), galactinol synthase (MdGOLS), raffinose synthase (MdRS), and stachyose synthase (MdSS), were generally more highly expressed during the early stage (Phase I) than the late stage (Phase II), while genes of enzymes catalyzing sugar consuming, such as fructose kinases (MdFRK), which catalyzes the production of fructose-6-P for entering glycolysis pathway, and cellulose synthases (MdCESA) and g galacturonosyltransferase (MdGAUT), which directs sugars to form cell wall components, was weakly expressed before 20 days after germination (DAG) but was dramatically activated thereafter (Figure 6). The results of qPCR are consistent with those of mRNA-seq, supporting this viewpoint (Figure S3). Most of the soluble sugars identified in the scion, including glucose, sucrose, galactose, and melibiose, increased dramatically at 20 DAG compared with 0 DAG and decreased towards 40 DAG. Raffinose, however, increased constantly in the scion. These changes in metabolic compounds and genes indicate that soluble sugars facilitate the formation of graft junctions, especially in the early phase.

### 2.7. Effects of Sugar Treatments on Graft Junction Formation

**Figure 7:**
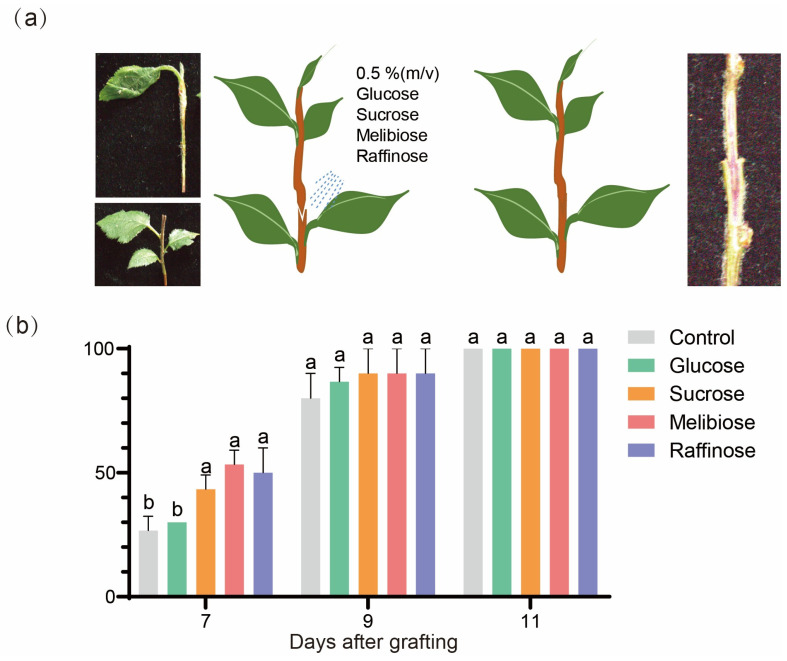
Effect of sugar treatments on graft healing of ‘Hanfu’/‘Hanfu’ micrograft. (**a**) The schematic drawing illustrates the measurement of the vascular connection rate. (**b**) Vascular connection rate. Different letters (a,b) indicate significant differences at the *p* < 0.05 level. Identical letters indicate no significant difference at this level.

The roles of sugars in graft junction formation were examined by spraying glucose, sucrose, melibiose, and raffinose (each at 0.5%) separately onto the graft junction at 0 DAG (Figure 7a). Grafts treated with 0.5% sucrose, melibiose, and raffinose had a better-developed callus, and the vascular connectivity rate in sugar treatments at 7 DAG was significantly higher than the control (*p* < 0.05 n = 3) (Figure 7b). This result proves that sugar availability is crucial for graft healing.

## 3. Discussion

### 3.1. Graft Junction Formation Occurs in Phases

Graft healing, which is crucial for graft success, is a complex development process affected by many factors, including the temperature [22], humidity, light intensity [23], photoperiod [20], grafting method, and, most importantly, genotypes of the scion and rootstock [2]. Despite significant differences in responses among various graft combinations [24], successful grafting processes are generally similar, involving similar successive graft junction formation events: callus proliferation, scion–stock callus bridging allowing cell-to-cell communication via plasmodesmata [25,26], vascular cell differentiation, and vascular reconnection between the stock and the scion [27,28]. In this study, callus formation, proliferation, and filling the gap between the scion and the stock (scion–stock callus bridging) could be observed in apple homograft within 20 DAG. It is worth noting that callus formation occurs chiefly from the scion. During this period, scion budbreak occurred, but vascular reconnection between the scion and the stock did not occur as the acid fuchsin dye could not move into the scion from the stock. After 20 DAG, the vascular connection rate increased rapidly, indicating increased vascular bundle reconnection. By 40 DAG, 100% of the graft junction samples showed vascular reconnection. Therefore, the graft junction formation process in ‘Hanfu’/‘Hanfu’ homograft can be divided into two distinctive phases: Phase I, from 0 to 20 DAG, which involves the formation and proliferation of callus, while Phase II, from 20 to 40 DAG, involves the re-establishment of vascular connection between the stock and the scion.

### 3.2. Scion vs. Stock Asymmetry Response to Grafting Is More Pronounced in Phase I

In previous studies, the rootstock and scion showed asymmetrical responses to grafting in Arabidopsis [29]. In *Pinus elliottii* homografts, certain plant hormones (e.g., auxin) and auxin-activated genes were differentially expressed between the scion and rootstock, implying that the auxin pathway played an important role in the graft healing process [30,31]. Physiological indexes such as sugar contents and their change patterns in grafted grapefruit showed asymmetry between the scion and the stock [32]. In our study, the asymmetrical response to grafting could be observed in callus formation, as callus formation occurs earlier in the scion. The transcriptome results also showed that there are more DEGs in the scions and the stock in Phase I than in Phase II and significant differences in gene expression profile between the rootstock and the scion in Phase I of graft healing. Those genes belonged to the brown and yellow modules in the WGCNA analysis results, and their expression patterns became more similar in Phase II. Both PCAs based on the transcriptome data and the metabolome data proved that asymmetry between the scion and the stock is more pronounced in Phase I than in Phase II, suggesting such asymmetry disappears with the vascular reconnection between the two graft parts. Our findings are consistent with those reported by Melnyk et al. (2018) in Arabidopsis, suggesting that the observed phenomena are not limited to a single species [28].

We selected DEGs and DEMs at three key time points (0, 20, and 40 DGA) for KEGG enrichment analysis. The results showed that these DEGs and DEMs were significantly enriched in sugar metabolic pathways. Considering that sugars, as important substrates in plant cell energy metabolism, are widely involved in cell growth and development and can also serve as monomers to polymerize into cellulose, starch, and other macromolecules, participating in cell construction, the differences in sugar metabolism may be related to the asymmetric expression patterns of the rootstock and scion during the grafting process. We found that various sugars, including glucose, fructose, stachyose, and melibiose, were most abundant at 20 DAG, the boundary between the early and late stages of graft healing, and significantly decreased after the vascular bundles were fully reconnected. Similarly, various sugar metabolic synthesis genes were highly expressed at 20DAG and then gradually decreased.

### 3.3. Graft Junction Formation Involves Shift in Sugar Metabolism and Is Dependent on Sugar Supply

KEGG enrichment of DEGs and DEMs display changes in differential pathways occurring between Phase I and Phase II as well as between the scion and the stock. However, the results highlight changes in sugar metabolism during graft junction formation. Most sugars increased drastically during Phase I, and buds decreased during Phase II. This is different from the result obtained in grapefruit grafts by Zhu [32], who found sugar contents in the stock had a trend of first decreasing and then increasing while sugars were relatively constant in the scion. Sugars play important roles in graft healing, as the process involves cell division and expansion dependent upon sugar supply [33]. The sharp increase in contents of most sugars detected in the scion and the stock during Phase I suggested that the sugars may be converted from reserves, for example, starch in the scion. Starch is broken down into glucose when the plant requires a large amount of energy, participating in energy metabolism pathways [34]. Starch reduction in the scion during healing has been observed in *Carya cathayensis* previously [35]. It is worth noting that the dynamic changes in starch are different in the scion and rootstock. In the grafting of cucumber onto pumpkin, the starch in the scion reaches its highest level at 1 DAG and then slowly decreases. In contrast, the starch in the rootstock steadily declines after grafting [36]. The proliferation and subsequent differentiation of callus cells involve a large amount of gene expression [37]. Genes of enzymes catalyzing sugar generation, such as amylase (MdAMY), invertase (MdINV), galactinol synthase (MdGS), raffinose synthase (MdRS), and stachyose synthase (MdSS), were generally more highly expressed during Phase I than Phase II, while genes of enzymes catalyzing sugar consumption, such as fructose kinases (MdSUS) which catalyze production of fructose-6-P for entering the glycolysis pathway, and cellulose synthases (MdCESA) and galacturonosyltransferase (MdGAUT) that direct sugars to form cell wall components, were weakly expressed in Phase I but activated dramatically in Phase II. These results indicate that sugars accumulate at the graft interface during callus formation and are consumed as they participate in cell wall synthesis following the reconnection of the vascular bundles.

### 3.4. Exogenous Sugars Promoted Graft Junction Formation

Exogenous application of hormones and sugar may reduce grafting incompatibility [38]. Carbohydrates are needed for the process of energy dissipation. According to the analysis of carbohydrate distribution, the energy consumed by callus cells in the early stage of healing may come from nearby tissues. The exogenous application of sugars may accelerate the grafting healing process. Previous studies have indicated that the application of 0.5% exogenous glucose on cucumber/pumpkin heterografts enhanced graft success, xylem reconnection, and the growth of grafted plants. This enhancement is more evident in etiolated seedlings where the sugar-signaling transduction gene TOR is inhibited [36]. Our results are similar to the previous research. The vascular connectivity rate of a 7 DAG wound treated with 0.5% sucrose, raffinose, and melibiose solution is much higher than that of other treatments, suggesting that the effect of sucrose, raffinose, and melibiose on plant grafting healing is reflected in promoting the healing process and accelerating healing, while 0.5% exogenous glucose were not significant in promoting these process in our study. Early studies have shown that sucrose is a favorable carbon source for callus development and can promote the development process of callus cells [39]. Sucrose is related to the adhesion of the grafting junction, and the success of grafting depends largely on the bonding strength of grafting tissues; stronger bonding strength is a prerequisite for successful grafting [5]. Raffinose was reported to rapidly accumulate in plants under drought stress, helping to conserve water by reducing transpiration from leaves. The overexpression of key enzymes involved in their synthesis, such as raffinose synthase (RS), can significantly enhance the drought tolerance of plants [40]. Melibiose is a disaccharide composed of galactose and glucose, and there are currently no reports on its specific role in the process of plant grafting. We assume that melibiose is hydrolyzed into glucose and galactose to promote grafting junction formation, as galactose is a primary component of cell wall polymers, glycolipids, and glycoproteins. According to our study, several sugars and treatments may indirectly affect the healing process by promoting the development of callus cells at the grafted junction and vascular reconnection.

## 4. Materials and Methods

### 4.1. Plant Materials and Grafting Methods

For analysis of the mechanism involved in graft junction formation in apple (Malus domestica), homograft was conducted using 1-year-old plants of the cultivar ‘Hanfu’ (Malus domestica). These plants were cultivated in the experimental fields of the China Agricultural University. Scions, with a stem diameter of approximately 1cm, from specific ‘Hanfu’ plants were grafted onto ‘Hanfu’ rootstocks of a similar diameter. The homograft plants were generated using the cleft grafting method on 20 March 2024. Graft junctions were randomly sampled at 0, 10, 20, 30, and 40 days after grafting (DAG) for further anatomic, transcriptomic, and metabolomic analyses.

For the experiment with sugar treatments, ‘Hanfu’ in vitro seedlings with stem diameter larger than 3 mm, which had been cultivated on the root forming mediate (MS + 1.0 mg/L IBA + 6.5 g/L agar pH = 5.8) for a month, were selected for micrografting using the cleft grafting method. The grafted plants were incubated under a photoperiod of 16 h of light and 8 h of darkness at a temperature of 25 °C.

### 4.2. Anatomical Observation for Timeline of Graft Junction Formation

A total of 30 samples of ‘Hanfu’/‘Hanfu’ graft junctions, sampled by cutting 5 cm below the grafting site at different grafting stages (0, 10, 20, 30, and 40 DAG), were randomly divided into three groups, with 10 samples in each group. The stock end of the sampled graft seedlings was submerged in 1% (*w*/*v*) acid fuchsin solution (sigma Sigma-Aldrich Corporation (St. Louis, MO, USA)) for 60 min, and then the graft junctions were vertically cut and observed under a stereoscope to check the distribution of the red dye across the graft joint. The vascular reconnection between the stock and the scion is indicated by the mobility of the dye from the stock to the scion. Then vascular reconnection rate at each stage could be calculated. Three biological replicates, each with 10 graft junctions, were set for each sampling time.

For further phenotype analysis, the graft junctions were fixed in formalin–alcohol–acetic acid (FAA) stationary liquid for 24 h, and samples were then infiltrated by paraffin and sectioned. The sections were stained with Safranin before microscopy examination and photography.

### 4.3. Transcriptome Sequencing and Analyses

To construct transcriptomic libraries, 20 graft junction samples, consisting of 0.25 cm from the grafting site of the stock seedling at 0 DAG, 0.25 cm from the scion, and 0.25 cm from the stock at the remaining grafting stages (10, 20, 30, 40 DAG) were collected (Figure S1). The samples were randomly divided into two groups, with ten samples in each group. Immediately after collection, the samples were frozen in liquid nitrogen and quickly ground into powder using a grinder. Total RNA was extracted with an EASYspin Plant Total RNA Rapid Extraction kit (Biomed Company, Beijing, China) according to the manufacturer’s instructions. Two biological replicates, each with 10 randomly pooled 10 graft junctions, were used to build RNA-seq libraries. RNA quality was assessed using a gel electrophoresis apparatus and nanodrop spectrophotometer. RNA with a concentration over 300 µg/mL, 28S rRNAs/18S rRNAs ratio over 2, and RNA integrity number over 6 were used to build the RNA-seq library. RNA sequencing was performed using the Illumina HiSeq 4000 platform (Illumina, Inc., San Diego, CA, USA) at Biomec Biotechnology., following standard protocols for library preparation and sequencing. The FPKM (Fragments Per Kilobase of transcript per Million fragments mapped) was calculated using featureCounts [41]. Differential expression analysis between groups was performed using DESeq2 [42]. DEGs for two groups were identified using *p*-value (<0.05) and |Log2FC| (≥1.0). To gain further insights into the graft junction formation, Kyoto Encyclopedia of Genes and Genomes (KEGG) enrichment analysis and Weighted Gene Co-expression Network Analysis (WGCNA) analysis were constructed using Ggplot2 and WGCNA packages in R script (version 4.4.2).

### 4.4. Metabolomic Analysis

Samples were collected as above for metabolomic analysis. After grinding into powder in liquid nitrogen, 1 g of the powder was weighed and suspended in 1.2 mL methanol (70%) extraction solution, then placed in a refrigerator at 4 °C overnight. The next day, the samples were removed from the refrigerator and centrifuged at 12,000 rpm for 10 min. The supernatant was carefully collected and filtered through a 0.22-μm microporous filter membrane for LC-MS/MS analysis. The LC-MS/MS analysis was conducted using a UHPLC-QE-MS system (Thermo Fisher Scientific, Waltham, MA, USA). Unsupervised multidimensional statistical analysis methods and orthogonal partial least squares discriminant analysis (OPLS-DA) were applied to enhance metabolomic differences between sample pairs [43]. DEMs for two groups were identified using *p*-value (<0.05) and VIP (≥1.0). Kyoto Encyclopedia of Genes and Genomes (KEGG) using Ggplot2 packages in R script.

### 4.5. RT–qPCR Validation

RT-qPCR was performed with the SYBR Green I Master kit (TIAN-GEN, Biotech Co., Ltd., Beijing, China) according to the manufacturer’s instructions. Primers used in this experiment are shown in Table S5. Apple actin gene was analyzed as an internal control using primer pair MdActin-F/MdActin-R. The qPCR procedure was conducted as follows: all reactions were incubated on 96-well plates at 95 °C for 10 min, followed by 40 cycles of 95 °C for 15 s, 60 °C for 32 s, then 95 °C for 15 s, 60 °C for 1 min, and 95 °C for 15 s. qPCR analysis was conducted in a StepOnePlus Real-Time PCR System (Thermo Fisher Scientific, Inc., USA).

### 4.6. Exogenous Soluble Sugars Treatments on Grafted Plantlets

To test the effect of soluble sugars on graft healing, exogenous glucose, sucrose, raffinose, or melibiose solution at a concentration of 0.5% (*w*/*v*) was sprayed on the graft junction of the micro-grafted plants at 0 DAG. Three biological replicates, each with 10 grafted plantlets, were used for every treatment. Vascular connection observation was carried out with acid fuchsin, as mentioned above.

## 5. Conclusions

The graft junction formation process consists of two phases: Phase I (0–20 days) involves callus formation and proliferation, while Phase II (20–40 days) is characterized by vascular reconnection. During Phase I, a significant asymmetry is observed between the scion and the rootstock, which gradually diminishes as vascular reconnection progresses. Sugar metabolism plays a crucial role in the formation of the graft junction, especially in the accumulation of sugars during callus formation and their subsequent consumption following vascular reconnection. The application of exogenous sugars (such as sucrose, raffinose, and melibiose) can significantly promote the formation and healing of the graft junction, indicating that sugar supply is essential for successful grafting.

## Data Availability

No data were used for the research described in this article. The datasets presented in this study can be found in online repositories.

## Supplementary Materials

The following supporting information can be downloaded at https://www.mdpi.com/article/10.3390/ijms26115290/s1.

## References

[1] Huang Y., Kong Q.S., Chen F., Bie Z.L. (2014). The history, current status and future prospects of vegetable grafting in China. Acta Horticulturae 1086, Proceedings of the I International Symposium on Vegetable Grafting, Wuhan, China, 17–21 March 2014.

[2] Mudge K., Janick J., Scofield S., Goldschmidt E.E. (2009). A history of grafting. Hortic. Rev..

[3] Tedesco S., Pina A., Fevereiro P., Kragler F. (2020). A phenotypic search on graft compatibility in grapevine. Agronomy.

[4] Goldschmidt E.E. (2014). Plant grafting: New mechanisms, evolutionary implications. Front. Plant Sci..

[5] Rasool A., Mansoor S., Bhat K.M., Hassan G.I., Baba T.R., Alyemeni M.N., Alsahli A.A., El-Serehy H.A., Paray B.A., Ahmad P. (2020). Mechanisms Underlying Graft Union Formation and Rootstock Scion Interaction in Horticultural Plants. Front. Plant Sci..

[6] Gur A., Samish R.M., Lifshitz E. (1968). Role of cyanogenic glycoside of quince in incompatibility between pear cultivars and quince rootstocks. Hortic. Res..

[7] Kollmann R., Yang S., Glockmann C. (1985). Studies on graft unions. II. Continuous and half plasmodesmata in different regions of the graft interface. Protoplasma.

[8] Kurotani K.I., Notaguchi M. (2021). Cell-to-cell connection in plant grafting—Molecular insights into symplasmic reconstruction. Plant Cell Physiol..

[9] Jeffree C.E., Yeoman M.M. (1983). Development of intercellular connections between opposing cells in a graft union. New Phytol..

[10] Flaishman M.A., Loginovsky K., Golobowich S., Lev-Yadun S. (2008). Arabidopsis thaliana as a model system for graft union development in homografts and heterografts. J. Plant Growth Regul..

[11] Cookson S.J., Moreno M.J.C., Hevin C., Mendome L.Z.N., Delrot S., Trossat-Magnin C., Ollat N. (2013). Graft union formation in grapevine induces transcriptional changes related to cell wall modification, wounding, hormone signalling, and secondary metabolism. J. Exp. Bot..

[12] Chen Z., Zhao J., Hu F., Qin Y., Wang X., Hu G. (2017). Transcriptome changes between compatible and incompatible graft combination of litchi chinensis by digital gene expression profile. Sci. Rep..

[13] Zheng B.S., Chu H.L., Jin S.H., Huang Y.J., Wang Z.J., Chen M., Huang J.Q. (2010). cDNA-AFLP analysis of gene expression in hickory (*Carya cathayensis*) during graft process. Tree Physiol..

[14] Thomas H., Van den Broeck L., Spurney R., Sozzani R., Frank M. (2021). Gene regulatory networks for compatible versus incompatible grafts identify a role for SlWOX4 during junction formation. Plant Cell.

[15] Mazur E., Benková E., Friml J. (2016). Vascular cambium regeneration and vessel formation in wounded inflorescence stems of Arabidopsis. Sci. Rep..

[16] Yin H., Yan B., Sun J., Jia P., Zhang Z., Yan X., Chai J., Ren Z., Zheng G., Liu H. (2012). Graft-union development: A delicate process that involves cell–cell communication between scion and stock for local auxin accumulation. J. Exp. Bot..

[17] Notaguchi M., Kurotani K.I., Sato Y., Tabata R., Kawakatsu Y., Okayasu K., Sawai Y., Okada R., Asahina M., Ichihashi Y. (2020). Cell-cell adhesion in plant grafting is facilitated by β-1,4-glucanases. Science.

[18] Wang Y., Kollmann R. (1996). Vascular differentiation in the graft union of in-vitro grafts with different compatibility.—Structural and functional aspects. J. Plant Physiol..

[19] Aloni R. (1980). Role of auxin and sucrose in the differentiation of sieve and tracheary elements in plant tissue cultures. Planta.

[20] Marsch-Martinez N., Franken J., Gonzalez-Aguilera K.L., Folter S., Angenent G.C., Alvarez-Buylla E.R. (2013). An efficient flat-surface collar-free grafting method for Arabidopsis thaliana seedlings. Plant Methods.

[21] Zhang L., Hu J., Han X., Li J., Gao Y., Richards C.M., Zhang C., Tian Y., Liu G., Gul H. (2019). A high-quality apple genome assembly reveals the association of a retrotransposon and red fruit colour. Nat. Commun..

[22] Serivichyaswat P.T., Bartusch K., Leso M., Musseau C., Iwase A., Chen Y., Sugimoto K., Quint M., Melnyk C.W. (2022). High temperature perception in leaves promotes vascular regeneration and graft formation in distant tissues. Development.

[23] Bartusch K., Trenner J., Melnyk C.W., Quint M. (2020). Cut and paste: Temperature-enhanced cotyledon micrografting for *Arabidopsis thaliana* seedlings. Plant Methods.

[24] Cookson S.J., Clemente Moreno M.J., Hevin C., Nyamba Mendome L.Z., Delrot S., Magnin N., Trossat-Magnin C., Ollat N. (2014). Heterografting with nonself rootstocks induces genes involved in stress responses at the graft interface when compared with autografted controls. J. Exp. Bot..

[25] Pina A., Errea P. (2005). A review of new advances in mechanism of graft compatibility–incompatibility. Sci. Hortic..

[26] Pina A., Errea P., Martens H.J. (2012). Graft union formation and cell-to-cell communication via plasmodesmata in compatible and incompatible stem unions of *Prunus* spp.. Sci. Hortic..

[27] Tiedemann R. (1989). Graft union development and symplastic phloem contact in the heterograft *Cucumis sativus* on *Cucurbita ficifolia*. J. Plant Physiol..

[28] Melnyk C.W., Schuster C., Leyser O., Meyerowitz E. (2015). A developmental framework for graft formation and vascular reconnection in Arabidopsis thaliana. Curr. Biol..

[29] Melnyk C.W., Gabel A., Hardcastle T.J., Robinson S., Miyashima S., Grosse I., Meyerowitz E.M. (2018). Transcriptome dynamics at Arabidopsis graft junctions reveal an intertissue recognition mechanism that activates vascular regeneration. Proc. Natl. Acad. Sci. USA.

[30] Harrison M.A., Pickard B.G. (1989). Auxin asymmetry during gravitropism by tomato hypocotyls. Plant Physiol..

[31] Liu Q., Wang X., Zhao Y., Xiao F., Yang Y. (2023). Transcriptome and physiological analyses reveal new insights into delayed incompatibility formed by interspecific grafting. Sci. Rep..

[32] Zhu T.F., Wang Y., Su X.L., Li X., Wang L.C. (2022). Physiological characters in the stock and the scion during graft healing and their relation to graft compatibility in grapefruit. Jiangsu Agric. Sci..

[33] Wang L., Ruan Y.L. (2013). Regulation of cell division and expansion by sugar and auxin signaling. Front. Plant Sci..

[34] Lopes M.A., Larkins B.A. (1993). Endosperm origin, development, and function. Plant Cell.

[35] Liu C., Hong J., Xia G., Huang J. (2009). Cytological observation on healing responses in grafting of *Carya cathayensis*. Sci. Silvae Sin..

[36] Miao L., Li Q., Sun T.S., Chai S., Wang C.L., Bai L.Q., Sun M.T., Li Y.S., Qin X., Zhang Z.H. (2021). Sugars promote graft union development in the heterograft of cucumber onto pumpkin. Hortic. Res..

[37] Hoermayer L., Montesinos J.C., Marhava P., Benková E., Yoshida S., Friml J. (2020). Wounding-induced changes in cellular pressure and localized auxin signalling spatially coordinate restorative divisions in roots. Proc. Natl. Acad. Sci. USA.

[38] Cohen R., Karni L., Aktas H., Edelstein M. (2010). Hormonal signaling in rootstock-scion interactions. Sci. Hortic..

[39] Button J. (1978). The Effects of some Carbohydrates on the Growth and Organization of Citrus Ovular Callus. Z. Für Pflanzenphysiol..

[40] Li F., Wang X.F. (2008). Advances in the Metabolism and Regulatory Key Enzymes of Raffinose Series Oligosaccharides in Plants. Acta Bot. Boreali-Occident. Sin..

[41] Liao Y., Smyth G.K., Shi W. (2014). featureCounts: An efficient general purpose program for assigning sequence reads to genomic features. Bioinformatics.

[42] Varet H., Brillet-Guéguen L., Coppée J.-Y., Dillies M.-A. (2016). SARTools: A DESeq2-and EdgeR-based R pipeline for comprehensive differential analysis of RNA-Seq data. PLoS ONE.

[43] Westerhuis J.A., van Velzen E.J., Hoefsloot H.C., Smilde A.K. (2010). Multivariate paired data analysis: Multilevel PLSDA versus OPLSDA. Metabolomics.

