# Supplementary material for: Integrative Analyses of Metabolome and Transcriptome Reveal Scion–Stock Asymmetry Reduction and Shift of Sugar Metabolism During Graft Junction Formation in Malus Domestica (‘Hanfu’) Homograft
Source: Int J Mol Sci. 2025 May 30;26(11):5290. doi: 10.3390/ijms26115290 (PMC12155446; doi:10.3390/ijms26115290)
Supplement: Supplementary file 1 [file ijms-26-05290-s001.zip › supplymentary figureS1-S3.pdf]

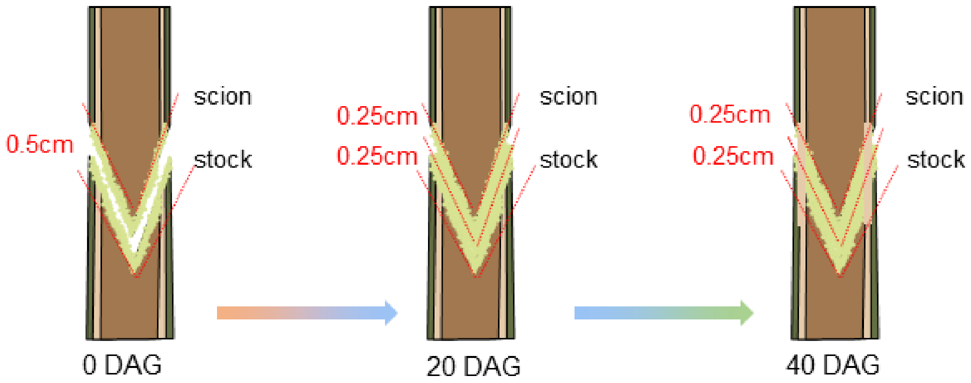

Figure S1. Schematic drawing of the scion and the stock parts sampled for transcriptomic and metabolomic analyses

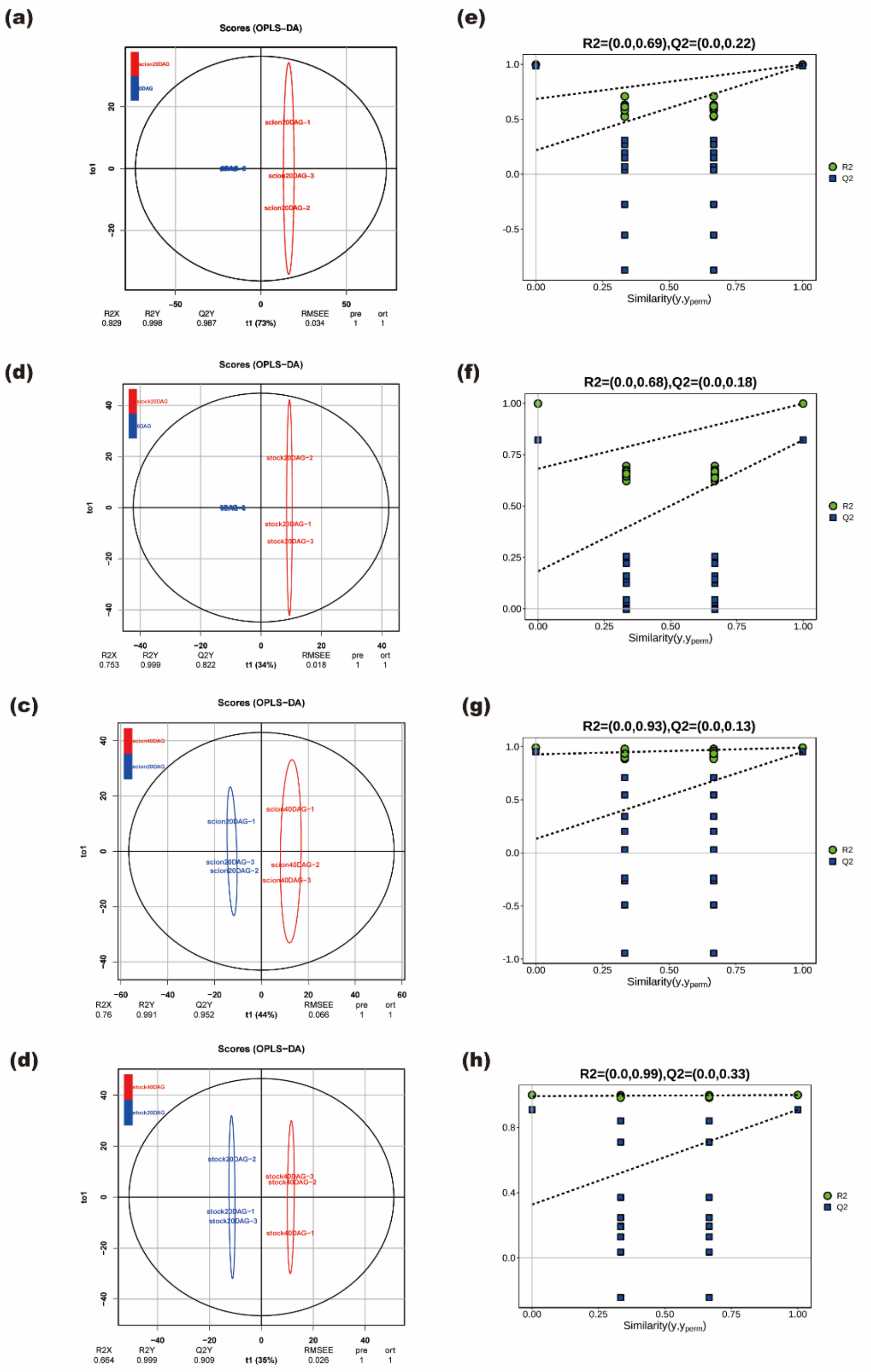

Figure S2 Orthogonal projection to latent structure-discriminant analysis (OPLS-DA) (a-d) and permutation test(f-h).

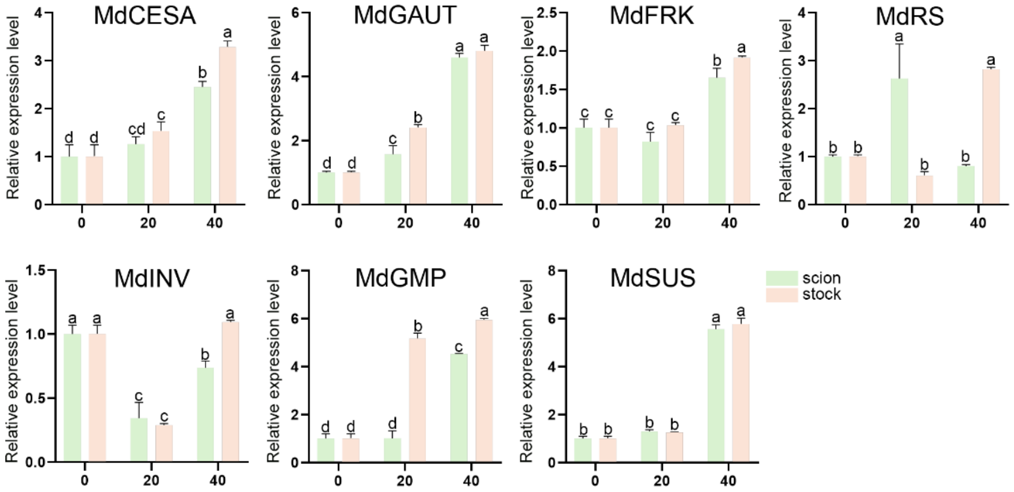

Figure S3 qRT-PCR validation of the expression profiles of seven key genes in sugar metabolism.
